# Construction of a radiogenomic association map of pancreatic ductal adenocarcinoma

**DOI:** 10.1186/s12885-023-10658-z

**Published:** 2023-02-27

**Authors:** Neema Jamshidi, Jayasuriya Senthilvelan, David W. Dawson, Timothy R. Donahue, Michael D. Kuo

**Affiliations:** 1 Department of Radiological Sciences, David Geffen School of Medicine, University of California, Los Angeles, 757 Westwood Ave, Suite 2125, Los Angeles, CA 90095 USA; 2 Jonsson Comprehensive Cancer Center, University of California, Los Angeles, CA USA; 3 Department of Pathology, University of California, Los Angeles, CA USA; 4 Department of Surgical Oncology, University of California, Los Angeles, CA USA; 5 Medical AI Laboratory Program, The University of Hong Kong, Hong Kong SAR, Hong Kong

**Keywords:** Pancreatic cancer, Radiogenomics, Network biology, Tumor enhancement, Non-invasive, Glycerophospholipid metabolism, Podosome assembly, Mitophagy, Infiltrative, TGF-β, Tumor-stroma interface, Contrast-enhanced CT, Transcriptome

## Abstract

**Background:**

Pancreatic adenocarcinoma (PDAC) persists as a malignancy with high morbidity and mortality that can benefit from new means to characterize and detect these tumors, such as radiogenomics. In order to address this gap in the literature, constructed a transcriptomic-CT radiogenomic (RG) map for PDAC.

**Methods:**

In this Institutional Review Board approved study, a cohort of subjects (*n* = 50) with gene expression profile data paired with histopathologically confirmed resectable or borderline resectable PDAC were identified. Studies with pre-operative contrast–enhanced CT images were independently assessed for a set of 88 predefined imaging features. Microarray gene expression profiling was then carried out on the histopathologically confirmed pancreatic adenocarcinomas and gene networks were constructed using Weighted Gene Correlation Network Analysis (WCGNA) (*n* = 37). Data were analyzed with bioinformatics analyses, multivariate regression-based methods, and Kaplan-Meier survival analyses.

**Results:**

Survival analyses identified multiple features of interest that were significantly associated with overall survival, including Tumor Height (*P* = 0.014), Tumor Contour (*P* = 0.033), Tumor-stroma Interface (*P* = 0.014), and the Tumor Enhancement Ratio (*P* = 0.047). Gene networks for these imaging features were then constructed using WCGNA and further annotated according to the Gene Ontology (GO) annotation framework for a biologically coherent interpretation of the imaging trait-associated gene networks, ultimately resulting in a PDAC RG CT-transcriptome map composed of 3 stage-independent imaging traits enriched in metabolic processes, telomerase activity, and podosome assembly (*P* < 0.05).

**Conclusions:**

A CT-transcriptomic RG map for PDAC composed of semantic and quantitative traits with associated biology processes predictive of overall survival, was constructed, that serves as a reference for further mechanistic studies for non-invasive phenotyping of pancreatic tumors.

**Supplementary Information:**

The online version contains supplementary material available at 10.1186/s12885-023-10658-z.

## Background

Pancreatic adenocarcinoma (PDAC) is an aggressive malignancy with a 5 year survival rate of less than 5%, in spite of extensive research and treatment efforts [1]. These difficulties highlight the need for identifying prognostic imaging biomarkers and more mechanistic studies, to elucidate the underlying biological mechanisms giving rise to observed clinical phenotypes [2].

Radiogenomics (RG) involves the integration of biological data with clinical imaging data and has been developed for the purpose of gaining insight into the relationships between imaging, cellular, and subcellular data [3]. It has successfully been employed to identify robust non-invasive biomarkers in multiple cancer types using different imaging modalities, including predicting complex patterns of gene expression, clinical prognosis, and treatment response [4–13]. The growth in the number of RG studies highlights the value and need for developing means for ‘non-invasive genotyping’ [14].

CT imaging is a critical diagnostic tool for determining treatment options including surgical feasibility and planning of PDAC. However, the ability to incorporate clinically relevant genomic as well as physiologic phenotypic information into a single non-invasive assay would be of great clinical and biological value. Semantic CT imaging features in other solid tumors have provided compelling prognostic information. However, cross-sectional imaging data can also capture information about molecular characteristics as well as the functional organization of cells and tissues. Clinical imaging phenotypes (or radiophenotypes), represent the summation of complex imaging features that account for physiologic and molecular interactions in the context of hierarchically organized cells, tissues, and organs. To date however multi-phase CT imaging with corresponding transcriptomic profiles in histopathologically confirmed PDAC in a well-defined population (i.e. surgically resectable) has not been available, thus RG studies for PDAC have not been feasible.

We hypothesized that a PDAC radiogenomic map could be developed that was associated with overall survival outcomes. We constructed a CT-transcriptomic signature associated with overall survival in borderline surgically resectable PDAC. Further bioinformatics analyses from whole genome transcriptomic profiling identified biological and processes associated with the map, thus setting the stage for a non-invasive, biologically meaningful RG map for PDAC.

## Methods

### Approach

Multi-phase CT scans in patients with histopathologically confirmed PDAC were interpreted for a set of pre-defined quantitative and semantic features. Survival analysis across the cohort was used to identify the imaging features predictive of survival. A RG map was then constructed by 1) constructing a transcriptomic association network from matched tumor samples, and 2) identifying the network hubs within the network that were significantly associated with these particular imaging features and survival outcome. Bioinformatics analyses were applied to the gene modules to characterize the biological processes and functions corresponding to the different imaging features in the RG map.

### Patients and materials

**Fig. 1:**
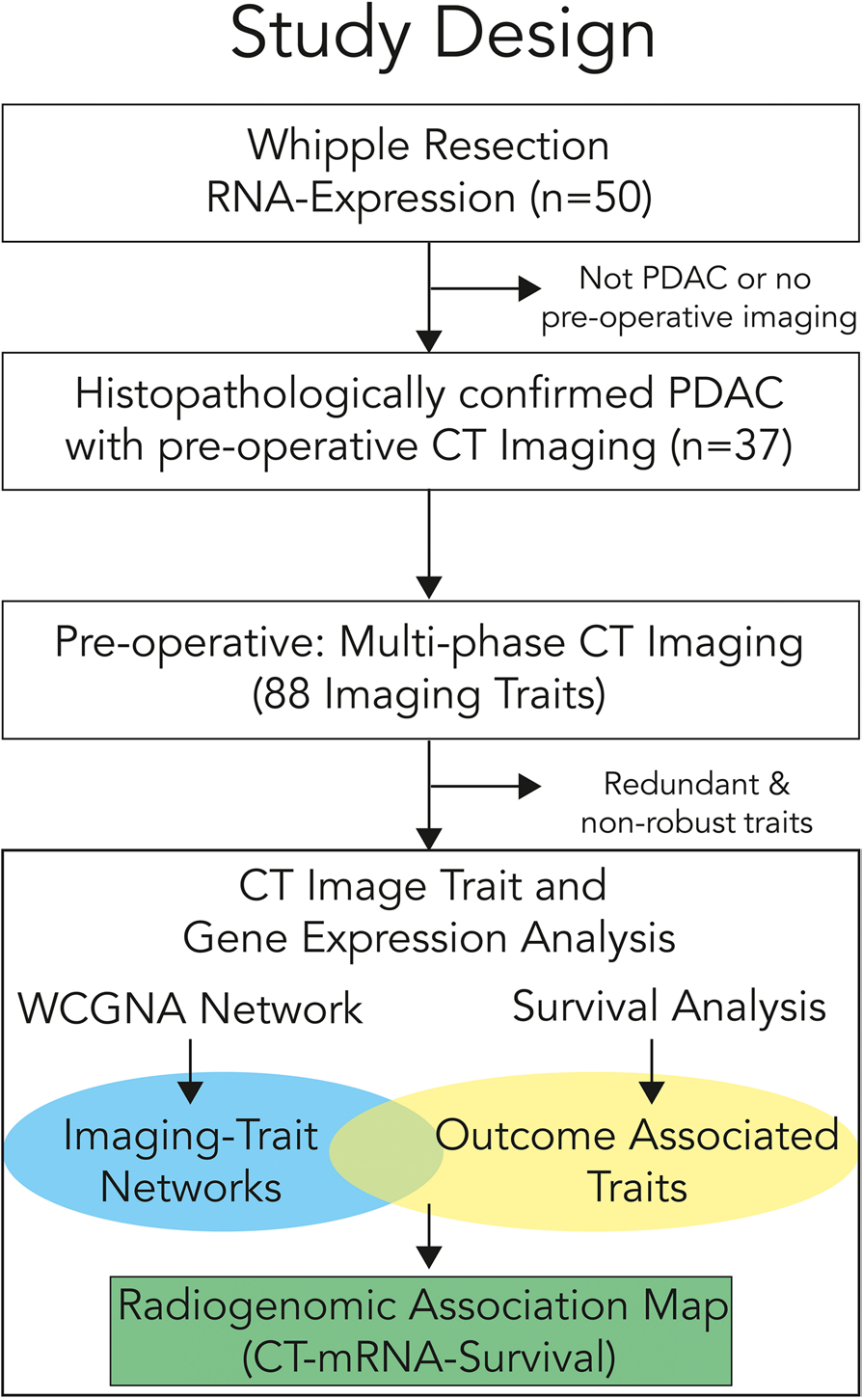
Schematic outline of the identification of cases with pre-operative multi-phase CT scans and corresponding tumor transcriptomic profiles in patients with pathologically confirmed PDAC

The cohort was identified from a retrospective set of sequential samples available from the surgical histopathology tissue bank collected between 2004 and 2009, with clinical outcomes, that were cross-referenced with PACS database image sets with pre-operative diagnostic contrast enhanced CT scans. Archived fresh frozen tissue samples, clinical data, and imaging studies obtained as part of routine clinical care were analyzed with approval by the local Institutional Review Board and in compliance with the Declaration of Helsinki. Data was acquired, including waiver of consent, following review and approval by the UCLA Institutional Review Board, Protocol Number 10–001869. The overall study design is shown in Fig. 1 and the cohort summary characteristics are described in Table 1.

**Table 1:** Cohort characteristics. 1-way ANOVA associations with survival were calculated for the cohort sub-groups (calculated *p*-values appear in the right-most column)

		n	*p*-value
**Age**	71 +/− 11 years		–
**Gender**			0.52
	Male	18	
	Female	19	
**Operation type**	Distal pancreatectomy	5	0.039
	Pylorus-preserving Whipple	10	
	Standard Whipple	22	
**Margin status**			0.57
	R0	33	
	R1	4	
**Stage**			0.089
(T2 versus T3)	T2N0M0	2	
	T2N2M0	6	
	T2N3M0	1	
	T3N0M0	11	
	T3N1M0	15	
	T3N2M0	1	
	T3N3M0	1	

### CT imaging and image feature analysis

Diagnostic contrast-enhanced computed tomographic scans were performed with 16-slice CT (Definition or Sensation, Siemens Healthcare) pre-operatively with 3 mm axial reconstructions and 5 mm coronal and axial reconstructions. Images were acquired with either dual phase or triphasic pancreas protocol. Studies were performed with dual phase fixed timing or bolus tracking protocols of iohexol 350 mg/ml at a rate of 3 mL/s. The former involved pre-contrast helically acquired images followed by pancreatic phase 35 seconds after triggering from aorta above the diaphragm and liver phase 10 seconds later. The latter involved pre-contrast helically acquired images 30 seconds following injection and liver phase 10 seconds later.

A collection of 88 pre-defined imaging traits [5, 9, 12, 15], composed of semantic as well as quantitative features reflecting structural, compositional, physiological and functional information, were used to assess the set of images (Supplementary Material, Section 1). Readers were trained on an initial set of 10 independent cases. All images were evaluated on a Digital Imaging and COmmunications in Medicine (DICOM) viewer workstation (OsiriX 64-bit), across the features in the image feature library, independently by two board certified radiologists (20 and 6 years experience, respectively), blinded to any clinical information regarding the patients. Discrepant semantic trait interpretations were subsequently resolved in joint consensus, blinded to any outcomes data. Quantitative trait measurements were averaged between the readers.

The set of 88 imaging features were reduced by filtering based upon significant (*P* < 0.05) associations with outcome (following the Benjamini-Hochberg correction for false discovery rate (FDR) < 0.25 cutoff). These imaging features constituted the basis for the radiogenomic map and network construction. Kaplan-Meier plot analyses for overall survival were performed for each of the imaging features that were identified by the significant correlations.

### Gene expression profiling

As described by Donahue et al. [16], tissue samples were snap frozen following surgical resection with confirmed tumor regions by hematoxylin and eosin staining or from tumors isolated from formalin-fixed paraffin-embedded (FFPE) tissue blocks and used as a validation cohort for quantitative PCR as described in [16]. Transcriptomic profiling was performed using Affymetrix HGU133 Plus 2 Arrays (Thermo Fisher Scientific) with normalization following Gene Chip Robust Multi-array Average (GCRMA) as implemented in R [17]. Following GCRMA normalization probes with less than 40% presence across all samples were removed [16]. Probes that mapped to multiple genes were resolved by selection of the probe with the largest calculated Manhattan norm of normalized expression values across the samples. Transcriptomic data are also available in the NCBI Gene Expression Omnibus Database (GEO accession: GSE32688).

### Bioinformatics and network analysis

Imaging feature to RNA expression network maps were constructed using the normalized transcriptomic profiles in conjunction with the significant imaging features identified in the image feature and survival analysis.

Weighted Gene Correlation Network Analysis (WCGNA) [18] was used to construct correlated gene networks for the set of tumor gene expression profiles. WGCNA was performed to cluster correlated genes into modules and find associations between modules and clinical traits. First, outlier genes were removed based on an examination of the clustering dendrogram of microarray genes. Due to the large number of probes (19,991), block-wise network construction and module detection was conducted. Genes were roughly clustered into blocks of size not more than 10,000 and soft-thresholding power of 5 was performed on each block. Highly-correlated modules among different blocks were then merged to create the final network [18]. Correlations between each module’s eigengene and every clinical trait were used to find modules suitable for in-depth analysis. Within these modules, genes with an expression pattern highly correlated to the clinical trait (gene significance) and eigengene (module membership) were recorded. The image-trait matrix was constructed as described above. Gene network to trait associations were evaluated for absolute values of the Pearson Correlation Coefficients (PCC) > 0.4. Subsequent annotation of biological functions and processes associated with gene network modules using Gene Ontology (GO) enrichment analysis was performed.

Comparisons between the WCGNA networks that were significantly associated with survival were compared with subtype grouping of PDAC by Bailey et al. [19] using official gene name identifiers per HUGO Gene Nomenclature Committee.

### Statistical analysis

Numerical and statistical calculations including the PCC (significance cutoff *P* < 0.05 and multi-hypotheses correction with Benjamini-Hochberg FDR < 0.25), Kaplan-Meier survival analysis with the Gehan-Wilcoxon test (significance defined as *P* < 0.05), and WCGNA were performed using R (http://www.r-project.org/), Statistica (StatSoft, Tulsa, OK), and Python. GO analysis was performed using the Gene Ontology enRIchment anaLysis (*P* < 0.05, FDR < 0.25) and visuaLizAtion (GOrilla) [20] web-api (http://cbl-gorilla.cs.technion.ac.il/).

## Results

The overall study design is outlined in Fig. 1; the initial target cohort (*n* = 50) was identified from a subset of chemotherapy naïve patients with histopathologically confirmed PDAC that were resected with acquisition of corresponding transcriptomic profiling of the tumors. This cohort was then further reduced based upon pre-operative imaging and the availability of clinical outcome data (*n* = 37).

### Patient characteristics

Across the entire cohort there were no significant differences among patients with respect to gender, margin status, or stage (Table 1). Distal pancreatectomies have been observed to have better survival and overall mortality than full Whipple surgeries [21, 22] and we observed this in our cohort as well (Table 1, *P* = 0.039).

### Imaging trait and survival analysis

Initial assessment by calculation of the pairwise PCC with overall survival identified five different imaging traits, including, Tumor height (Trait 3.3), Endo- versus Exo-phytic Tumor (Trait 19), Tumor Contour (Trait 20), Tumor-stroma Interface (Trait 32), and the Enhancement Ratio (Trait 59). Subsequent Kaplan-Meier analyses reduced this list to four traits that had significant association with survival including Tumor height, Tumor Contour, Tumor-stroma Interface, and the Enhancement Ratio (Trait 59).

**Fig. 2:**
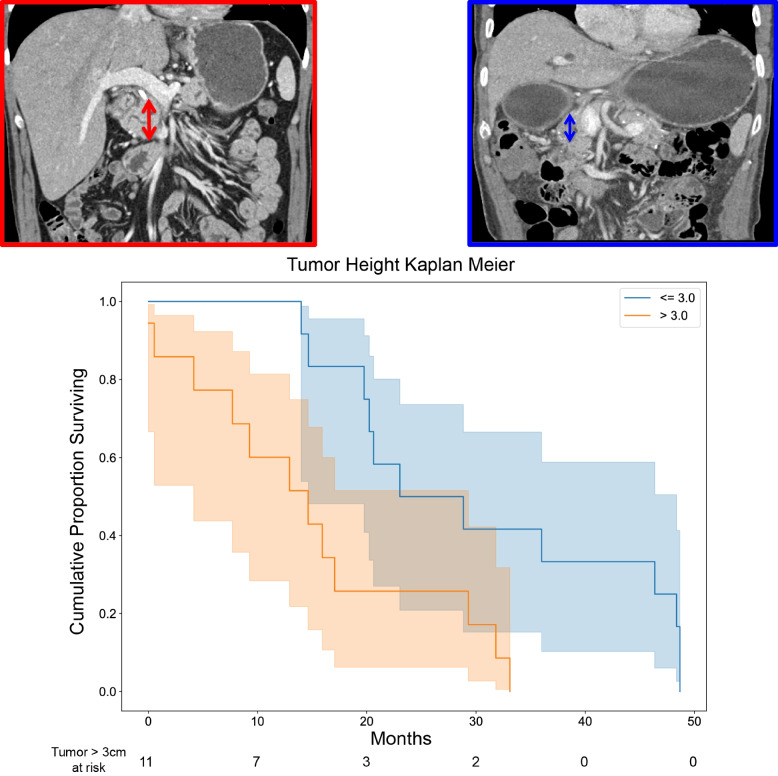
Kaplan-Meier plot of overall survival as a function of tumor height (coronal plane measurement). Example images for tumor height less than 3 cm and greater than 3 cm are shown in the left hand sub-panels next to the survival plot (solid blue solid and red lines, respectively) (*P* = 0.014)

**Fig. 3:**
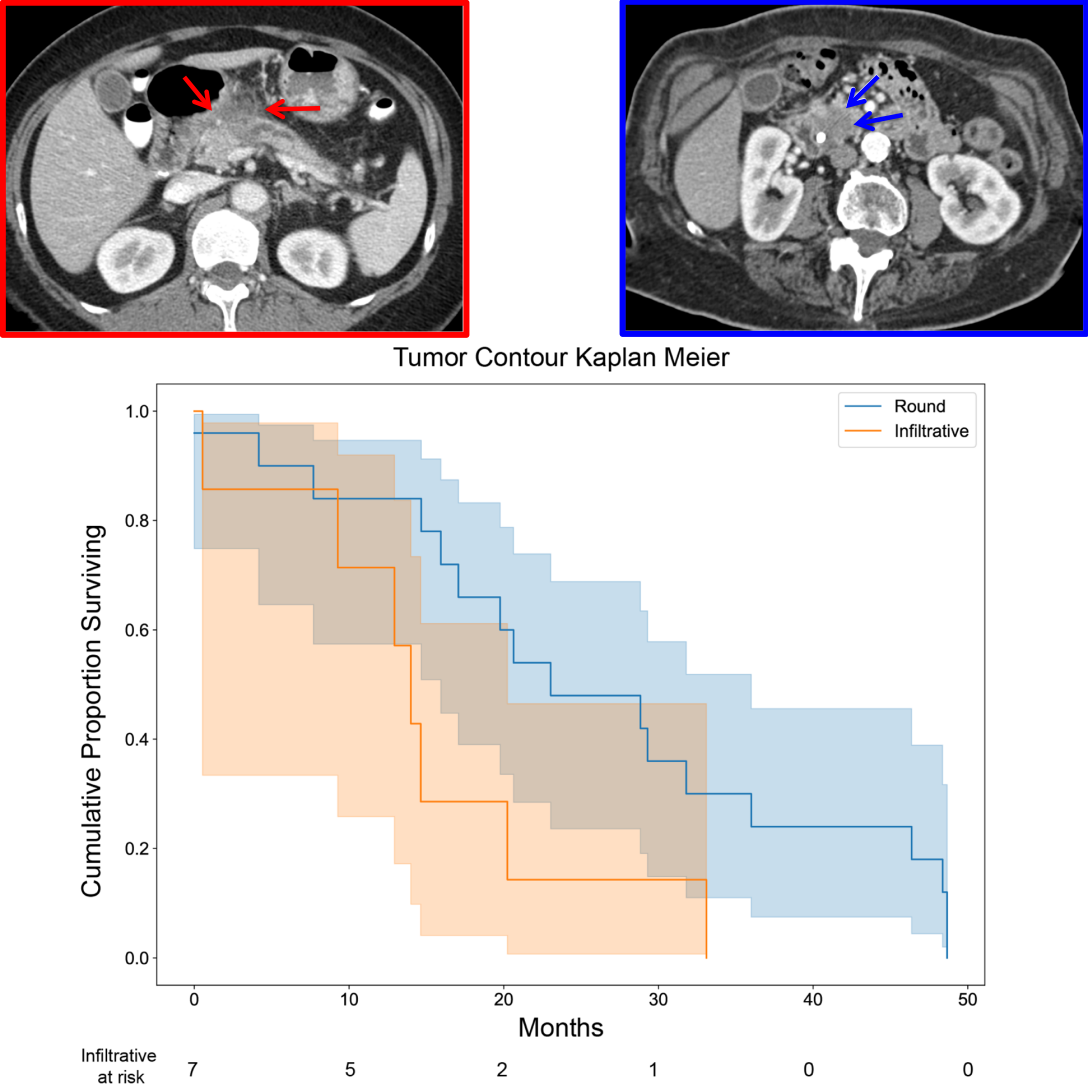
Kaplan-Meier plot of overall survival as a function of the Tumor Contour trait. Example images for round and infiltrative are shown in the left hand sub-panels next to the survival plot (solid blue and red lines, respectively) (*P* = 0.033)

**Fig. 4:**
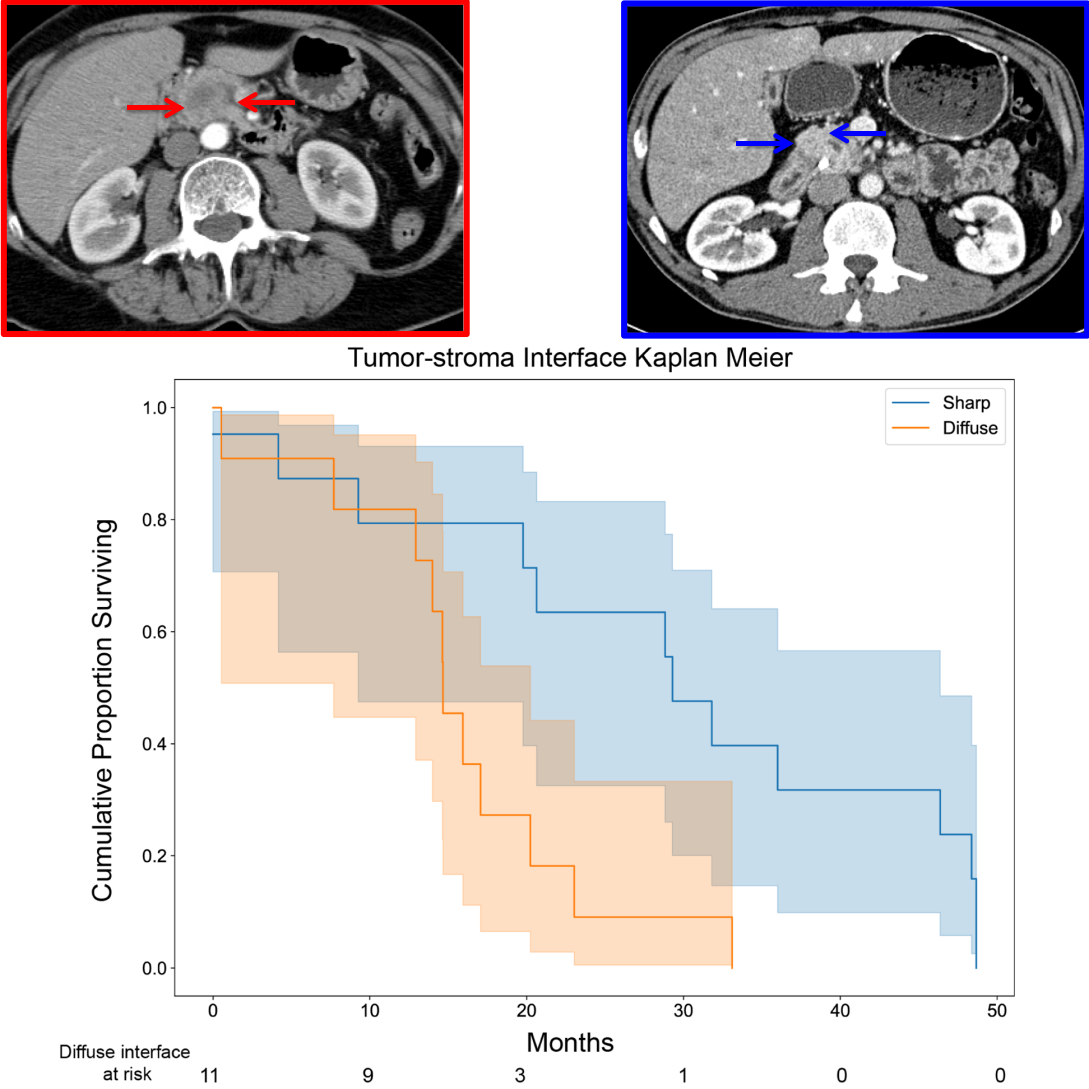
Kaplan-Meier plot of overall survival as a function of the Tumor-stroma Interface trait. Example images for the sharp and diffuse interfaces are shown in the left hand sub-panels next to the survival plot (solid blue and red lines, respectively) (*P* = 0.014)

**Fig. 5:**
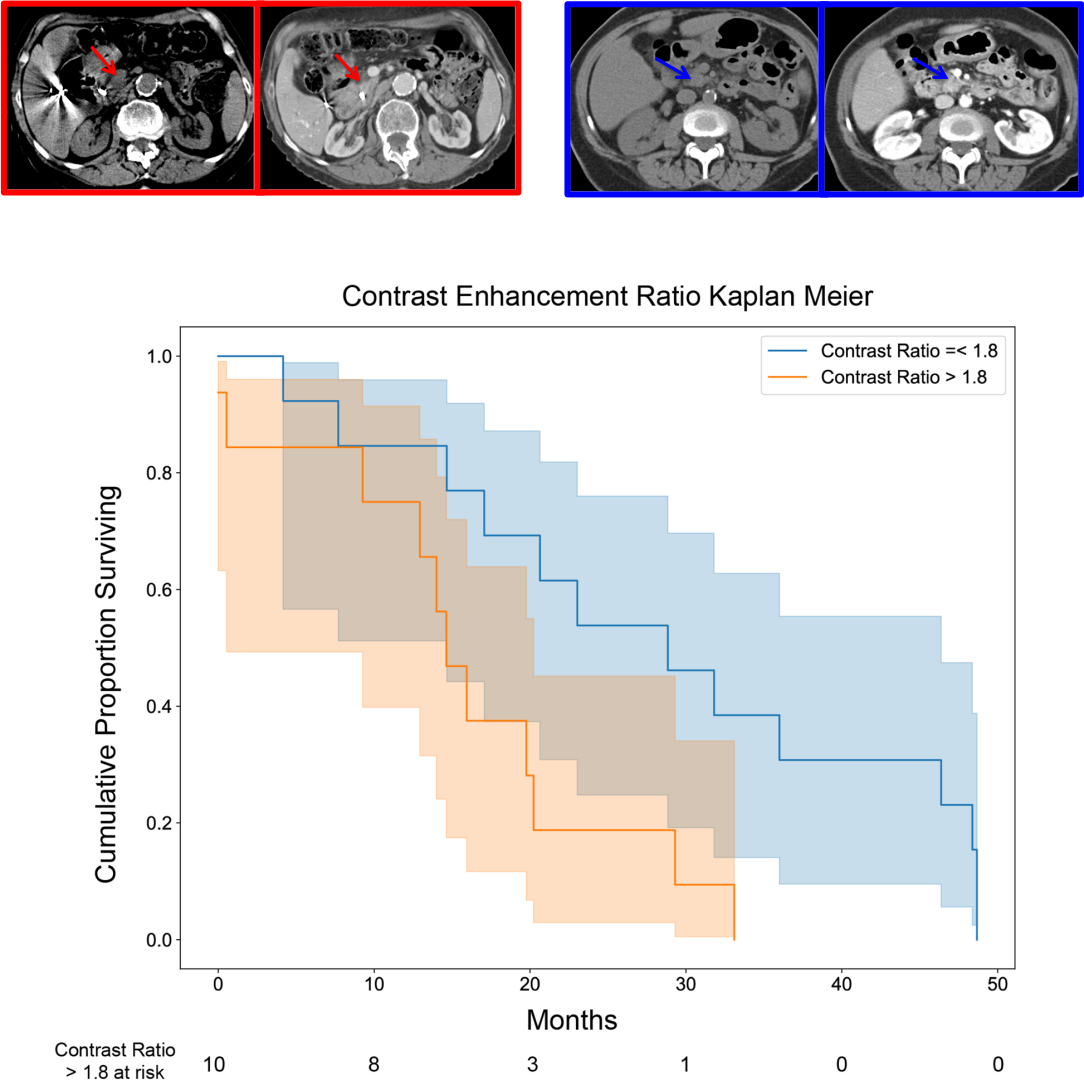
Kaplan-Meier plot of overall survival for Enhancement Ratio quantitative trait. Example images for values less than or equal to 1.8 and greater than 1.8 are shown in the left hand sub-panels next to the survival plot (solid blue and red lines, respectively) (*P* = 0.047)

Specifically for each of the aforementioned imaging features, larger tumor height (measurement in the coronal plane) (Fig. 2), infiltrative Tumor Contour (Fig. 3), diffuse Tumor-stroma Interface (Fig. 4), and decreased Enhancement Ratio (less than 1.8) (Fig. 5) were each independently, significantly associated with lower overall survival.

### RG analysis

WCGNA was used to construct gene networks associated with the sub-set of imaging traits that were significantly associated with overall survival. These networks were further characterized using GO enrichment analysis to identify the predominant biological processes, if any, that were associated with the imaging traits (Supplementary Material, Section 2).

Interestingly, three of the four independent imaging features were enriched in specific biological processes or functions. These GO biologically annotated processes were non-overlapping, consistent with the independence of the imaging features.

Tumor height was associated with 29 different GO functions and processes (*P* < 0.05). Common biological themes that arose in multiple processes included phospholipid metabolism (glycerophospholipid metabolism, phosphatidylinositol metabolism, and related redox metabolic processes) and mitochondrial structural/metabolic changes (mitophagy and mitochondrion-derived vesicle formation) (Table 2).

**Table 2:** GO enrichment annotations and associations for the gene network associated with increased tumor height; these processes collectively involve metabolic (anabolic and catabolic) processes

GO term	Description	P-value	FDR q-value	Enrichment
GO:0004497	monooxygenase activity	3.61E-05	1.68E-01	3.35
GO:0099073	mitochondrion-derived vesicle	1.08E-04	2.12E-01	20.98
GO:1990247	N6-methyladenosine-containing RNA binding	1.60E-04	3.73E-01	11.99
GO:0046854	phosphatidylinositol phosphorylation	1.73E-04	1.00E+00	3.96
GO:0005839	proteasome core complex	1.83E-04	1.80E-01	6.62
GO:0055076	transition metal ion homeostasis	2.02E-04	1.00E+00	2.77
GO:0008654	phospholipid biosynthetic process	2.70E-04	1.00E+00	2.15
GO:0010499	proteasomal ubiquitin-independent protein catabolic process	3.38E-04	1.00E+00	5.99
GO:0009889	regulation of biosynthetic process	4.03E-04	1.00E+00	1.21
GO:0061734	parkin-mediated stimulation of mitophagy in response to mitochondrial depolarization	4.16E-04	1.00E+00	15.73
GO:0031326	regulation of cellular biosynthetic process	4.24E-04	9.33E-01	1.22
GO:0009952	anterior/posterior pattern specification	4.31E-04	8.29E-01	2.51
GO:0046486	glycerolipid metabolic process	4.65E-04	7.96E-01	1.86
GO:0016723	oxidoreductase activity, oxidizing metal ions, NAD or NADP as acceptor	5.33E-04	8.29E-01	9.32
GO:0006650	glycerophospholipid metabolic process	5.56E-04	8.56E-01	1.93
GO:1900076	regulation of cellular response to insulin stimulus	5.70E-04	7.98E-01	3.2
GO:0006644	phospholipid metabolic process	5.88E-04	7.54E-01	1.84
GO:2000112	regulation of cellular macromolecule biosynthetic process	6.50E-04	7.69E-01	1.22
GO:0016722	oxidoreductase activity, oxidizing metal ions	6.84E-04	7.98E-01	6.56
GO:0003700	DNA-binding transcription factor activity	6.87E-04	6.41E-01	1.41
GO:0010556	regulation of macromolecule biosynthetic process	7.58E-04	8.33E-01	1.21
GO:0016712	oxidoreductase activity, acting on paired donors, with incorporation or reduction of molecular oxygen, reduced flavin or flavoprotein as one donor, and incorporation of one atom of oxygen	7.85E-04	6.11E-01	4.45
GO:0046916	cellular transition metal ion homeostasis	8.32E-04	8.54E-01	2.75
GO:0046626	regulation of insulin receptor signaling pathway	8.39E-04	8.07E-01	3.28
GO:0098780	response to mitochondrial depolarisation	9.31E-04	8.43E-01	6.17
GO:0016705	oxidoreductase activity, acting on paired donors, with incorporation or reduction of molecular oxygen	9.42E-04	6.29E-01	2.35
GO:0045017	glycerolipid biosynthetic process	9.61E-04	8.22E-01	2.08
GO:0046906	tetrapyrrole binding	9.66E-04	5.64E-01	2.41
GO:0046474	glycerophospholipid biosynthetic process	9.80E-04	7.94E-01	2.12

The Tumor Contour (infiltrative versus round) was associated with 4 GO processes (*P* < 0.05), all related to telomerase activity, TGF-β signaling, and their inverse relationship (Table 3).

**Table 3:** GO enrichment annotations for the WCGNA gene network associated with the Tumor Contour trait, notable for increased telomerase activity and down regulation of TGF-β. Some of these biological processes have been independently described in the literatures, including TGF-β induced telomerase-dependent pancreatic tumor cell cycle arrest [23] and TGF-β receptor mediated telomerase inhibition and telomere shortening associated with cellular senescence [24]. Further, the reciprocal relationship between telomerase activity and TGF-β activity are consistent with reports in the literature

GO term	Description	P value	FDR q-value	Enrichment
GO:0003720	telomerase activity	3.50E-04	8.17E-01	68.17
GO:0003964	RNA-directed DNA polymerase activity	3.50E-04	1.00E+00	68.17
GO:0030512	negative regulation of transforming growth factor beta receptor signaling pathway	6.05E-04	1.00E+00	10.36
GO:1903845	negative regulation of cellular response to transforming growth factor beta stimulus	6.96E-04	1.00E+00	9.98

The Tumor-stroma Interface trait (diffuse versus sharp) was associated with 16 GO functions and processes (*P* < 0.05). Interestingly most of these are related to sugar metabolism (D-xylose, xylulose, xylulose 5-phosphate, uronic acid, glucuronate, etc) and podosome assembly (Table 4).

**Table 4:** GO enrichment annotations for the gene network associated with the Tumor-stroma Interface trait. Various anabolic and catabolic processes are identified in conjunction with spliceosome changes and podosome assembly

GO term	Description	P value	FDR q-value	Enrichment
GO:0042732	D-xylose metabolic process	3.35E-05	5.16E-01	197.47
GO:0005997	xylulose metabolic process	6.69E-05	5.15E-01	148.1
GO:0051167	xylulose 5-phosphate metabolic process	1.11E-04	2.85E-01	118.48
GO:1901159	xylulose 5-phosphate biosynthetic process	1.11E-04	3.43E-01	118.48
GO:0019640	glucuronate catabolic process to xylulose 5-phosphate	1.11E-04	4.28E-01	118.48
GO:0006064	glucuronate catabolic process	1.11E-04	5.71E-01	118.48
GO:0005861	troponin complex	3.10E-04	6.09E-01	74.05
GO:0071803	positive regulation of podosome assembly	3.97E-04	8.73E-01	65.82
GO:0050866	negative regulation of cell activation	4.16E-04	8.00E-01	7.92
GO:0006928	movement of cell or subcellular component	5.54E-04	9.48E-01	2.8
GO:0071006	U2-type catalytic step 1 spliceosome	7.23E-04	4.75E-01	49.37
GO:0071012	catalytic step 1 spliceosome	7.23E-04	7.12E-01	49.37
GO:0019585	glucuronate metabolic process	7.23E-04	9.28E-01	49.37
GO:0071801	regulation of podosome assembly	7.23E-04	1.00E+00	49.37
GO:0006063	uronic acid metabolic process	7.23E-04	1.00E+00	49.37
GO:0019321	pentose metabolic process	8.53E-04	1.00E+00	45.57

We considered the possibility that the tumor-stroma interface was associated with tumor resection margin status, however there was no significant association; the majority of cases were R0 and within the R0 cohort 43% were diffuse and 57% were sharp).

We also considered the possibility of associations between single genes to these traits (Supplementary Material, Section 3). No associations were identified that satisfied the PCC and Kaplan-Meier analysis for the specific significance and FDR cutoffs.

Given the efforts in recent years to subtype PDAC in terms of transcriptomic profiles, we evaluate the concordance of the subtypes as described by Bailey et al. [19] (in terms of the Reactome and WGCNA classifications). With respect to Reactome group classification, the Tumor Height had the most overlap with the Squamous subtype as well as the Immunogenic subtype to a lesser degree (Supplementary Material, Section 5). The grey WGCNA gene set had the highest degree of overlap with Tumor Height, Tumor Contour, and Tumor-stroma Interface, however it also had the most genes in the reference set.

## Discussion

The development of PDAC and the subsequent clinical course is complex and multifactorial. Investigating how sub-cellular alterations relate to tissue and organ level changes has the potential to improve the understanding of the histopathological progression of the disease as well as non-invasive means for detecting and phenotyping the tumors. Our systematic evaluation of CT imaging features and traits in conjunction with a systems analysis of transcriptomic profiles has resulted in construction of a RG map for PDAC (three semantic features and one quantitative). Subsequent analysis explored the potential biological significance of these features through construction of a transcriptomic association map that was interrogated using GO analysis. Three different traits that were each significantly associated with different survival outcomes as well as different biological processes/pathways. Most of the biological processes that were identified are consistent with biological processes in cancer development and progression that have been described in the literature [25].

The GO analysis identified telomerase activity and TGF-β signaling to be associated with the Tumor Contour (infiltrative versus round) trait. There is a reciprocal relationship between telomerase activity and TGF-β activity. Telomerase reverse transcriptase (TERT) has a broad yet specific range of effects that regulate normal function, but have also been implicated in tumor progression and malignant transformation [26, 27]. Specifically, in PDAC, TGF-β can induce telomerase-dependent pancreatic tumor cell cycle arrest by stimulation of the G1/S transition via TERT over-expression [23]. Additionally, TGF-β receptor mediated telomerase inhibition, telomere shortening and breast cancer cell senescence [24]. It is interesting to note that the reciprocal relationship between TGF-β activity and telomerase activity that has been described in the literature has also been recapitulated in the Tumor Contour gene network annotation.

Our analyses also revealed enrichment of multiple metabolic biological processes in addition to podosome formation associated with increased infiltrativeness of the tumor (Tumor-stroma Interface). Interestingly, podosome formation was been associated with increased invasiveness in pancreatic cancer [28–30].

Finally, tumor height was significantly associated with biological processed related to phospholipid metabolism, redox processes, and structural mitochondrial changes. The implication and association with metabolism suggests a further role for systems biology characterization of tumors of varying size.

There are multiple limitations to this study that should be recognized. This was a retrospective (with prospective analysis of the data), single institution study that is limited by the cohort size. Additionally, as observed with other RG studies, most of the significant traits involve semantic features; this can be a challenge for subsequent applications targeting automated image analysis. Given the uniqueness of the dataset and novelty of the presented work, there were not any published studies that we were aware of to provide independent validation against. Overall however, this serves a valuable dataset for identifying a reference CT-transcriptomic RG PDAC map that can aid in the design and development of further prospective studies.

Moreover the peripheral, enhancement characteristics were associated with survival outcomes; this is a similar theme to other RG markers in solid tumors of other organs, including glioblastoma multiforme, hepatocellular carcinoma, invasive breast carcinoma, and clear cell renal cell carcinoma. Thus, characterization of the peripheral contrast enhancement may reflect a general class of cross-sectional imaging biomarkers and warrants exploration in future studies.

Efforts for automated and machine-learning approaches to learn the semantic features will help with image-processing, throughput, and consistency in RG assessment of CT studies. Radiomic studies will clearly be helpful in this space [31], particularly with regards to standardization and harmonization of studies. While there have been some recent efforts with CT texture analysis in PDAC [32], we are not aware of any formal RG studies. This manuscript focuses on identification of predefined semantic features associated with overall outcomes with subsequent elucidation of the associated biological processes via bioinformatics GO analysis. But identifying potential biological features associated with imaging traits move one step closer towards non-invasive molecular characterization of these tumors.

As more knowledge has been gained about the different genomic alterations that underlie the development of PDAC, the biological microenvironment has been recognized to be an important factor in understanding tumor aggressiveness, the potential for immunosuppression, and thus influencing efficacy of drug treatment strategies [33]. The association between particular imaging features (e.g. infiltrativeness) with TGF-β, for example, suggests a potential link between the macroscopic CT features and the microscopic alterations of the microenvironment.

Although there was some overlap between the gene modules and transcriptomic subtypes that have been described for PDAC [19], the DICE scores were not significant. The lack of an association is not necessarily surprising, since the potential search space for creating RG predictors is very large [3] and the transcriptomic PDAC subtypes are based upon general trends across the entire cell, have evolved over the past decade, and do not presently have a unique, consensus definition [19, 34, 35]. We have shown that an RG approach can be used to construct an imaging predictor based upon transcriptomic signatures in renal cell carcinoma [4], and this may be possible for PDAC as well in the future.

There has also been increased appreciation in the literature of the multi-factorial aspects involved in the development and progression of tumors, notably that multiple functional changes need to occur for a tumor to grow and invade surrounding tissue [25, 36–39]. Thus it is interesting that the imaging features identified to be significantly associated with overall survival are associated with different, but potentially complementary, biological processes.

## Conclusions

The development and progression of pancreatic cancer is not a one-dimensional problem, and the different manifestations of the tumor generally result from multiple molecular and genomic processes. Thus, it is necessary to try to characterize different types of molecular measurements for the same tumor in order to identify some of the links among the datasets. Combining a systems biology analysis of transcriptomic alterations in PDAC with contrast enhanced CT imaging may lend insight into the biological processes underlying the nuanced differences in tumor appearance for patients with similar tumors, but different clinical courses.

Collectively, initial findings identify genomic biomarkers that have been confirmed in the literature and are consistent with assessment of gene ontology pathway enrichment. While the current sample size is a limitation of this study, the support of some of the findings with previously identified genes in the literature is encouraging. As this prospective study progress, the correlations will be assessed and updated as further follow-up data from the patients is obtained. A radiogenomic map of pancreatic adenocarcinoma suggests a role for contrast-enhanced CT as a predictive, non-invasive imaging surrogate reflecting the underlying genomics of the tumor.

## Supplementary Information

**Additional file 1.**

## Data Availability

All data generated or analyzed during this study are included in this article and the supplementary information.

## Abbreviations

CT: Computed Tomography
DICOM: Digital Imaging and COmmunications in Medicine
FDR: False discovery rate
FFPE: Formalin-fixed paraffin-embedded
GCRMA: Gene Chip Robust Multi-array Average
GO: Gene Ontology
GORILLA: Gene Ontology enRIchment anaLysis and visuaLizAtion
PACS: Picture Archiving and Communications System
PCC: Pearson Correlation Coefficient
PDAC: Pancreatic ductal adenocarcinoma
RG: Radiogenomics
TERT: Telomerase Reverse Transcriptase
TGF-β: Transforming Growth Factor-β
WGCNA: Weighted Gene Correlation Network Analysis
