# Supplementary material for: Construction of a radiogenomic association map of pancreatic ductal adenocarcinoma
Source: BMC Cancer. 2023 Feb 27;23:189. doi: 10.1186/s12885-023-10658-z (PMC9969670; doi:10.1186/s12885-023-10658-z)
Supplement: Supplementary file 1 — Additional file 1. [file 12885_2023_10658_MOESM1_ESM.pdf]

SUPPLEMENTARY INFORMATION

Further details and information in support of the main text.

1. Definitions of the pre-defined imaging traits.
2. Further details of the Gene Ontology enrichment analysis, including GO enrichment maps with processes/features that were significantly associated with specific Imaging Traits.
3. Summary of the single gene analysis.
4. Imaging trait matrix
5. PDAC subtypes
6. Summary of the PDAC-RG CT-Transcriptome Map.
7. References

1. Trait Names and Definitions (see also (1-3) for further details)

Trait Number	Trait Names	Trait Values (or Units if quantitative)
3.1	Tumor Length	cm
3.2	Tumor Width	cm
3.3	Tumor Height	cm
3.4	Tumor Approximate Volume	cm^3
4	Location	1= uncinate; 2=head; 3=neck; 4=body; 5=tail
5	Fat Infiltration	Yes: 1; No: 0
6	Texture	cystic: 1; solid: 2; mixed: 3
7	Bowel Invasion	Yes: 1; No: 0
8	Vein Thrombosis	Yes: 1; No: 0
9	Regional Lymph Nodes	None: 0; 1: 1-3; 2: 4-7; 3: > 7
10	Hemorrhage	Yes: 1; No: 0
11	Calcifications	Yes: 1; No: 0
12	Enhancement Pattern	homogenous: 1; heterogeneous: 2; peripheral: 3
13	Pre-contrast (least dense area)	HU
14	Pre-contrast (most dense area; no Ca++)	HU
15	Post-contrast (least dense area)	HU
16	Post-contrast (most dense area; no Ca++)	HU
17	Percent Necrosis	0: 0-25%; 1: 25-50%; 2: 50-75%; 3: 75-100%
18	Tumor Margins	Smooth: 1; Irregular: 2 (at least 50% of margin)
19	Endophytic vs Exophytic	Endophytic: 1; Exophytic expansile: 2
20	Tumor Contour	Round: 1; Infiltrative: 2 (50% of margin)
21	Pancreatic Duct	Non-dilated: 0; Dilated: 1
22	Common Bile Duct	Non-dilated: 0; Dilated: 1; Stented: 2
23	Pancreatic Atrophy	Yes: 1; No: 0
24	Distant Metastasis	Yes: 1; No: 0
25	Cysts or Stones	Yes: 1; No: 0
26	Tumor Transition Zone	Sharp: 1; Broad: 2
27	Contrast to Necrosis Ratio	Mild: 1; Moderate: 2; Severe: 3
28	Tumor Heterogeneity (pre-contrast)	Mild: 1; Moderate: 2; Severe: 3
29	Tumor Heterogeneity (post-contrast)	Mild: 1; Moderate: 2; Severe: 3
30	Tumor Capsule	Absent: 0; Present: 1
31	Tumor Enhancing Rind	No rind: 0; Enhancing rind: 1
32	Tumor-stroma Interface	Sharp: 1; Diffuse: 2
33	Enhancement Halo	Yes: 1; No: 0
34	Internal Arteries	Yes: 1; No: 0
35	Internal Septations	Yes: 1; No: 0

36	Arteries in Areas of Necrosis	Yes: 1; No: 0
37	Vessels Around Edge of Tumor	Yes: 1; No: 0
38	Tumor Nodularity	None: 0; Mild: 1; Moderate: 2; Severe: 3
39.1	Transition Zone (pre-contrast; point 1)	HU
39.2	Transition Zone (pre-contrast; point 2)	HU
39.3	Transition Zone (pre-contrast; point 3)	HU
39.4	Transition Zone (pre-contrast; point 4)	HU
40.1	Transition Zone (post-contrast; point 1)	HU
40.2	Transition Zone (post-contrast; point 2)	HU
40.3	Transition Zone (post-contrast; point 3)	HU
40.4	Transition Zone (post-contrast; point 4)	HU
41.1	Normal Tissue (pre-contrast)	Normal Tissue HU (pre-contrast)
41.2	Normal Tissue (post-contrast	Normal Tissue HU (post-contrast)
42	Tumor Thrombosis	Yes: 1; No: 0
43	Aorta (pre-contrast)	HU
44	Aorta (post-contrast; venous phase)	HU
44.1	Aorta (post minus pre)	HU
45	Portal Vein (pre-contrast)	HU
46	Portal Vein (post-contrast; venous)	HU
46.1.	Portal Vein (post minus pre)	HU
47	Touching SMA	Yes: 1; No: 0
48	Touching SMV	Yes: 1; No: 0
49	Direct Solid Organ Invasion	Yes: 1; No: 0
50	Heterogeneity Range (pre-contrast)	Difference: Pre-contrast (most dense area; no Ca++) minus Pre-contrast least dense area; no Ca++)
51	Heterogeneity Range (post-contrast)	Difference: Post-contrast (most dense area; no Ca++) minus Post-contrast least dense area; no Ca++)
52	Heterogeneity Enhancement Index 1	Ratio: Heterogeneity Range (post-contrast) to Heterogeneity Range (pre-contrast
53	Heterogeneity Enhancement Index 2	Ratio: Heterogeneity Enhancement Index 1 to tumor volume
54	Tumor Range Heterogeneity Index 1	Difference: Post-contrast (most dense area; no Ca++) minus Pre-contrast least dense area; no Ca++)
55	Tumor Range Heterogeneity Index 2	Ratio: Post-contrast (most dense area; no Ca++) to Pre-contrast least dense area; no Ca++)
56	Tumor Range Heterogeneity Index 3	Ratio: Tumor Range Heterogeneity Index 1 to tumor volume
57	Tumor Range Heterogeneity Index 4	Ratio: Tumor Range Heterogeneity Index 2 to tumor volume
58	Contrast Ratio 1	Ratio: Post-contrast (least dense area) to Pre-contrast (least dense area)
59	Contrast Ratio 2	Ratio: Post-contrast (most dense area; no Ca++) to Pre-contrast (most dense area; no Ca++)

60	Contrast Ratio 3	Ratio: Pre-contrast (most dense area; no Ca++) to Pre-contrast (least dense area; no Ca++)
61	Contrast Ratio 4	Ratio: Post-contrast (most dense area; no Ca++) to Post-contrast (least dense area; no Ca++)
62	Contrast Ratio 5	Ratio of Ratios: (Post-contrast (most dense area; no Ca++) to Post-contrast least dense area; no Ca++)) to Pre-contrast (most dense area; no Ca++) to Pre-contrast least dense area; no Ca++)))
63	Slope pancreas	slope pancreas (ref)
64	Slope pancreas (point 1)	slope pancreas (point 1)
65	Slope pancreas (point 2)	slope pancreas (point 2)
66	Slope pancreas (point 3)	slope pancreas (point 3)
67	Slope pancreas (point 4)	slope pancreas (point 4)
68	Slope pancreas difference	HU
69	Slope pancreas 5	slope of ((point 4-point 3)+(point 3-point 2)+(point 2-point 1))/(5+5+5)
70	Slope pancreas 6	slope of [((4-3)+(3-2)+(2-1))/(5+5+5)]/slope of nl panc
71	Slope pancreas 7	slope of [(((4-3)+(3-2)+(2-1))/(5+5+5)]/slope of aorta
72	Slope pancreas 8	slope of [(((4-3)+(3-2)+(2-1))/(5+5+5)]/slope of portal vein
73	Slope pancreas 9	slope of [(((4-3)+(3-2)+(2-1))/(5+5+5)]/slope of nl panc/tumor volume
74	Slope pancreas 10	slope of [((c-b)+(b-a))/(5+5+5)]/slope of aorta normalized by tumor volume
75	Slope pancreas 11	slope of [((4-3)+(3-2)+(2-1))/(5+5+5)]/slope of portal vein normalized by tumor volume
76	Slope pancreas 12	slope of precontrast phase [((point4-3)+(3-2)+(2-1))]/(5+5+5)
77	Slope pancreas 13	slope of post-contrast phase [((point4-3)+(3-2)+(2-1))]/(5+5+5)
78	Slope pancreas 14	slope of post contrast phase/slope of precontrast phase

2. Gene ontology enrichment analysis

GO analysis of WCGNA modules as described in Methods. Maps of GO processes are provided only for those modules that meet the significance criteria ($P < 0.05$ and correlation cutoff exceeds 95% interval).

Imaging Trait: Tumor-stroma Interface GO enrichment map

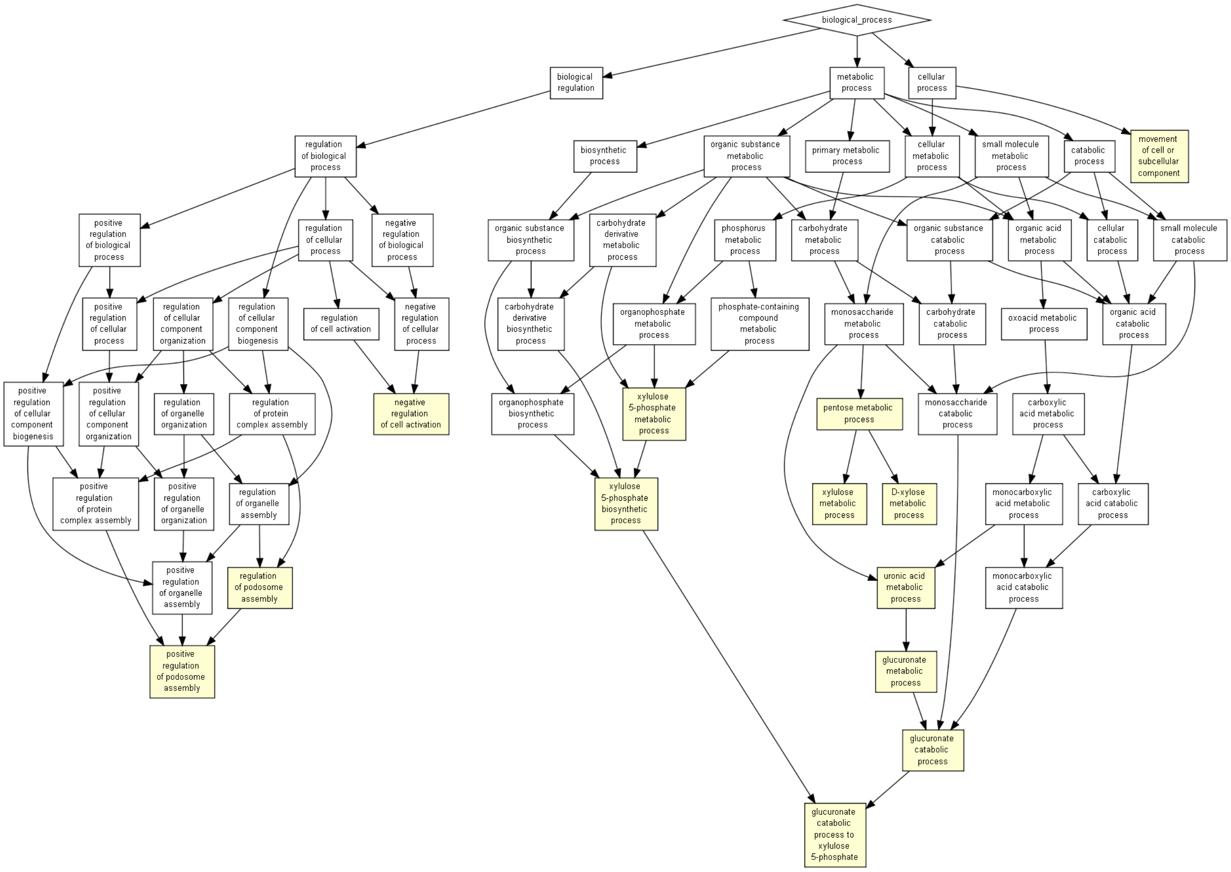

Imaging Trait: Tumor Contour GO enrichment map

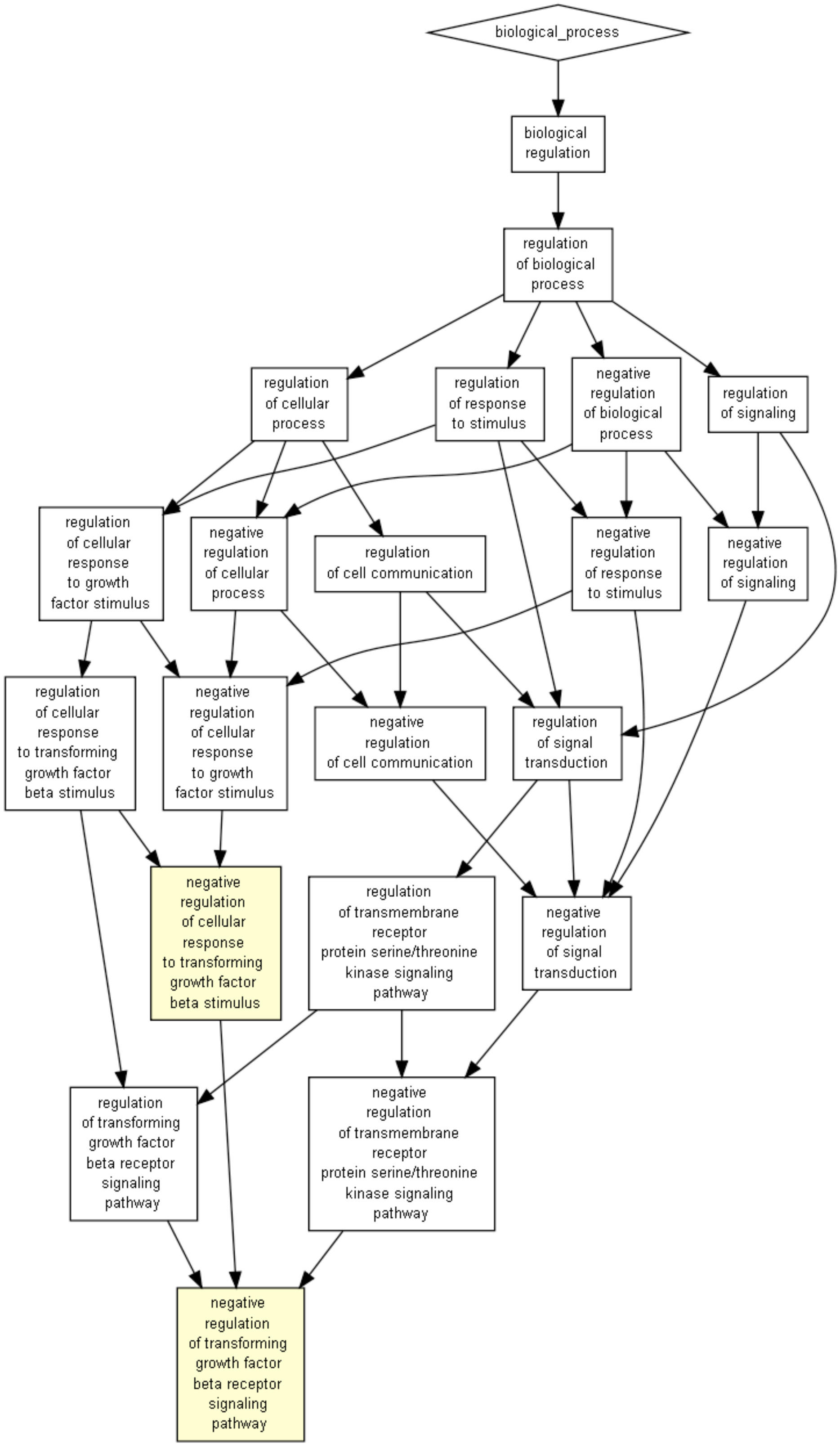

Imaging Trait: Tumor Height GO enrichment map

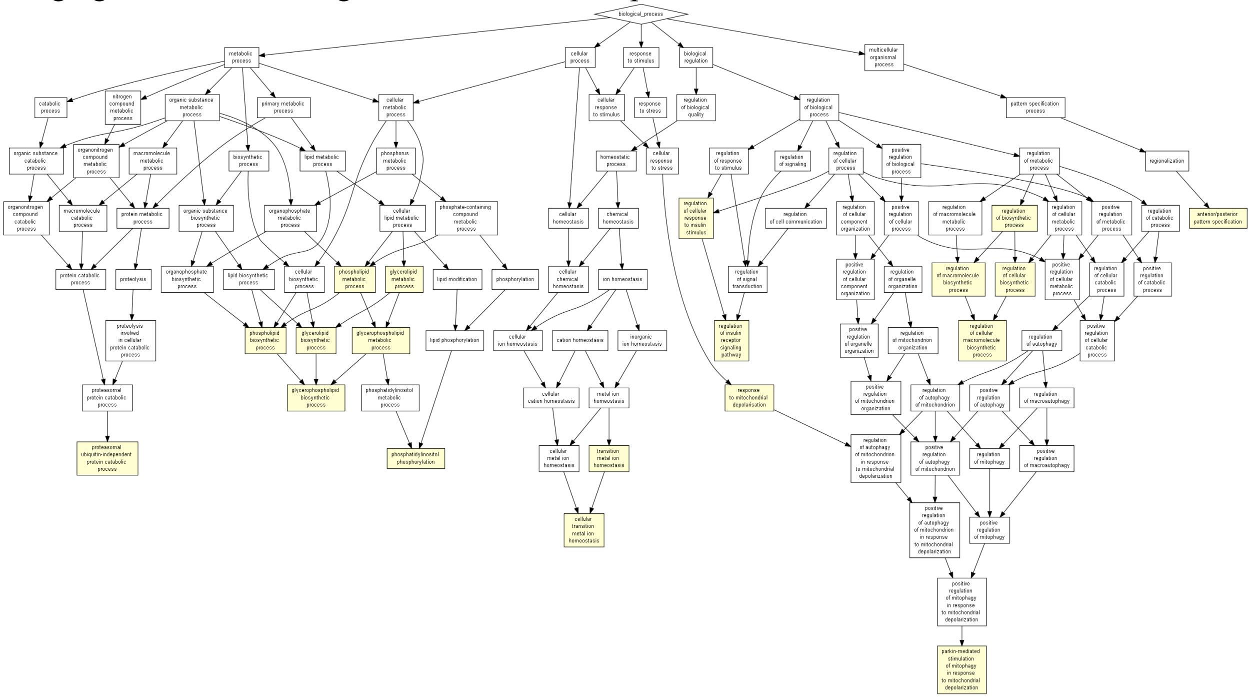

3. Single gene analysis

Gene expression profiling was carried out on the resected tumor tissue. Following normalization of the mRNA and miRNA datasets, variance filtering at 60% cutoff was performed. Resampling statistics (1000 permutations, sampling the traits without replacement), were used to determine the statistically significant cutoffs for Spearman correlation coefficient based associations between imaging traits and genes ($p<0.05$) (1, 4). Correlation coefficients were calculated between the imaging traits and mRNA expression profiles as well as the imaging traits and miRNA profiles, independently. Identification of the traits that correlated with survival was performed by calculating the Spearman correlation coefficient for traits with survival and selecting those with a coefficient of 0.4 and higher (or -0.4 and lower), for further analysis. For the subset of traits that satisfied this criteria, patients were stratified into two classes and cumulative survival analysis was carried out. In this prospective study, survival data was available for 19 of the 30 patients at the time of analysis.

miRNA to imaging trait association maps showed agreement with the literature, for example the negative associative between miR-22 (tumor suppressor) (5) and Trait26 (Tumor Transition Zone). Let-7a (pancreatic cancer suppressor) was found to correlate positively with the Trait29 (Timuor Heterogeneity Trait) and negatively with Venous Thrombosis (Trait8), which was also consistent with the survival correlations. Similarly, some of the gene correlations associated with imaging traits showed consistency with the literature. For example, tumor heterogeneity and the degree of necrosis were found to correlate with KRAS oncogene mRNA expression. Gene-ontology pathway analysis (Go-Miner (6)) of tumor heterogeneity demonstrated enrichment of molecular functions that were also consistent with this, such as DNA binding transcription factor activity, chromatin binding.

We considered the possiblity that individual "driver genes" may have associations with imaging features. Five genes that have been described (or associated) with suscpetibiltiy to developing PDAC or association with prognosis were selected and evaluated independently. Individual gene associations: Evaluated gene associations with pancreatic cancer driver genes, KRAS, TP53, CDNK2A, SMAD4, and BRCA2**.** A significant association was identified with Trait26 (Pearson Correlation Coefficient 0.56), however as noted in the main text, there was no significant survival stratification for Trait26.

4. Imaging Trait Matrix (Column headings correspond to the Trait numbering in Section 1)

3.3 (split at 3.0cm)	20	32 (split non-infiltrative vs infiltrative)	59 (split less than 1.8 vs greater than 1.8)
0	2	1	0
0	1	0	1
0	2	1	0
1	2	1	1
0	2	1	0
1	1	0	0
1	2	1	0
1	1	0	0
1	2	1	0
1	1	1	1
1	1	0	0
0	1	0	0
0	1	0	1

1	1	1	0
0	1	1	1
1	2	1	1
0	1	0	1
0	1	0	1
0	1	0	1
0	1	0	1
0	1	1	1
1	1	0	0
0	2	0	1
1	1	0	1
0	1	0	1
1	1	0	1
0	1	1	0
0	1	0	1
0	1	0	1
1	1	0	1
1	1	1	1
1	2	1	0
1	1	0	0
1	2	1	0
1	2	0	0
0	2	1	1
0	1	0	1

5. PDAC Subtypes

In recent years there have been efforts to subtype PDAC in terms of genomics and transcriptomics. We evaluated the concordance between subtypes described by Bailey et al (7) and the WCGNA networks associated with the traits of interest identified in this study. While there were subsets of overlapping genes identified, the DICE scores were all <0.1, thus there were not associations that were deemed to be significant. WGCNA comparison resulted in 208 genes overlapping with Tumor Height and the Bailey WGCNA grey network module, 24 genes overlapping between Tumor Contour and the Bailey WGCNA grey network module, and 15 genes overlapping between the Tumor-stroma Interface and the Bailey grey network module.

We then also performed a comparison with overlap between Reactome reaction sets and the gene sets identified with the four traits of interest.

Tumor Height:

Reactome Group 1 matched: APOBEC1, GALNT3, SRC, GNA11, INPP1, PLA2G16, CDX2, SIGIRR, ARHGEF12, FBP1, INSR, MNX1, CCNB3

Reactome Group 2 matched: SART1, EYA1, GNA12, LAMC2, ITGA6

Reactome Group 3 matched: EFEMP1, MAPK11, GLI2, GRIP1, WASF1, MYL9, MYH9, RASAL2, GPC6, BNIP2, LRP12, IGFBP5, GLI3, CDH2, TEAD1, WT1, PBX3, THBS3, DDR2, ADAM12, NR3C1, BOC, LATS2, COL15A1, ETS1, COL10A1

Reactome Group 4 matched: FANCE, BUB1B, MCM3, AURKA, TACC3, ARHGAP11A, PCNA, HIST1H2AB, NCAPD2, CCNE1, HMBS

Reactome Group 5 matched: NUP98, HNRNPC, CCT5, PSMB3, ETF1

Reactome Group 6 matched: RNF144B

Reactome Group 7 matched: ST8SIA4, ARSB, CLEC7A, PLA2G7

Reactome Group 8 matched: ARHGEF6, CD8A, ACAP1

Tumor Contour:

Reactome Group 1 matched: TGOLN2, LLGL2, ZNF786, CYP2C19, NRG3, GCH1

Reactome Group 3 matched: ASPN, SKI, MFAP5

Tumor-stroma Interface:

Reactome Group 3 matched: MEF2C, MSN, LAMB2

Summary of Reactome Group matching

Reactome Group	Tumor Height	Trait 20	Trait 32	Squamous	ADEX	Progenitor	Immunogenic
1	++	+				++	++
2	+			++			
3	++	+	+	++			
4	++			++			
5	+			++			
6							++
7	+						++
8							++
9					++		
10					++		

RG PDAC CT-Transcriptome Map

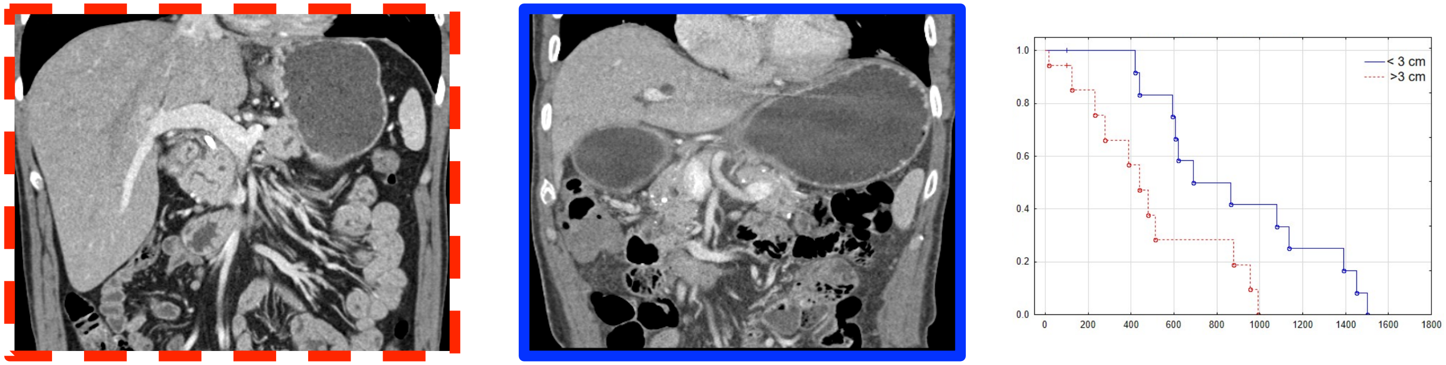

Glyerophospholipid Metabolism, Mitophagy, Biosynthetic processes

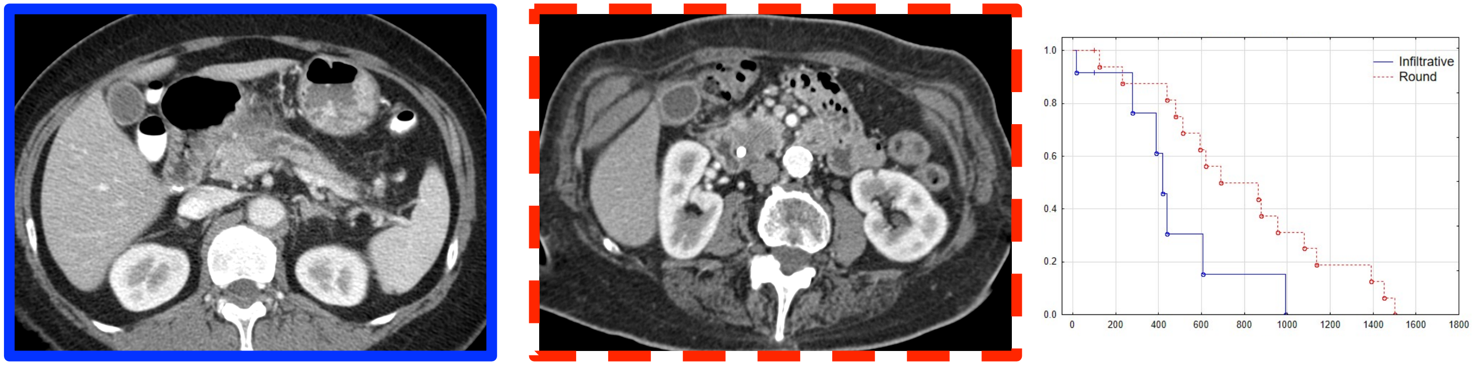

TGF-β Inhibition & Telomerase activity

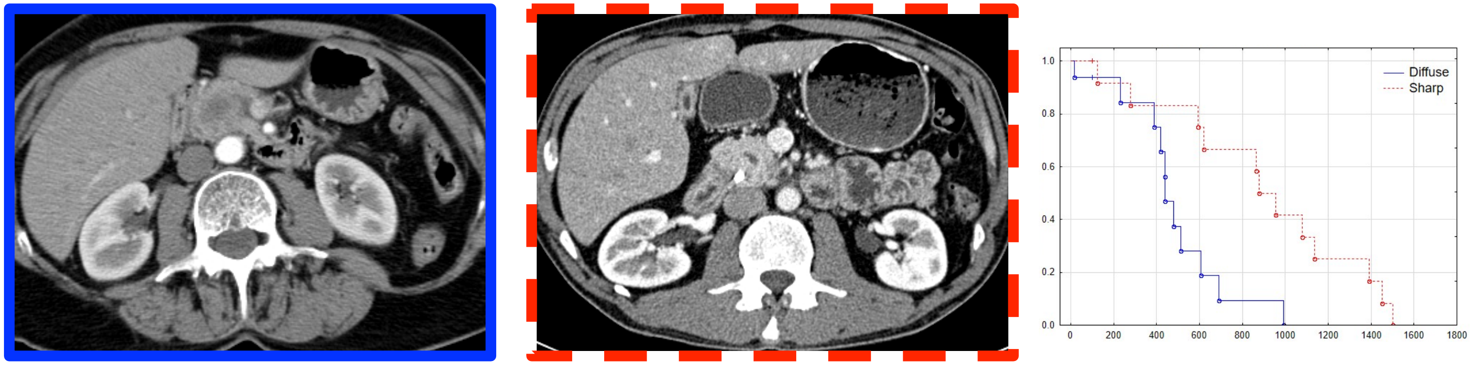

Sugar Metabolism, Podosome Assembly

7. REFERENCES

1. Diehn M, Nardini C, Wang DS, McGovern S, Jayaraman M, Liang Y, et al. Identification of noninvasive imaging surrogates for brain tumor gene-expression modules. *Proceedings of the National Academy of Sciences of the United States of America.* 2008;105(13):5213-8.
2. Jamshidi N, Jonasch E, Zapala M, Korn RL, Aganovic L, Zhao H, et al. The Radiogenomic Risk Score: Construction of a Prognostic Quantitative, Noninvasive Image-based Molecular Assay for Renal Cell Carcinoma. *Radiology.* 2015;277(1):114-23.
3. Segal E, Sirlin CB, Ooi C, Adler AS, Gollub J, Chen X, et al. Decoding global gene expression programs in liver cancer by noninvasive imaging. *Nature biotechnology.* 2007;25(6):675-80.
4. Sheskin D. *Handbook of parametric and nonparametric statistical procedures.* Boca Raton: Chapman and Hall/CRC; 2004.
5. Park JY, Helm J, Coppola D, Kim D, Malafa M, and Kim SJ. MicroRNAs in pancreatic ductal adenocarcinoma. *World J Gastroenterol.* 2011;17(7):817-27.
6. Zeeberg BR, Feng W, Wang G, Wang MD, Fojo AT, Sunshine M, et al. GoMiner: a resource for biological interpretation of genomic and proteomic data. *Genome Biol.* 2003;4(4):R28.
7. Bailey P, Chang DK, Nones K, Johns AL, Patch AM, Gingras MC, et al. Genomic analyses identify molecular subtypes of pancreatic cancer. *Nature.* 2016;531(7592):47-52.
